# An Apology for the Nerves, or Their Influence and Importance in Health and Disease

**Published:** 1845-04

**Authors:** 


### An Apology for the Nerves, or their Influence and Importance in Health and Disease. By Sir *George Lefevre*, M.D. late Physician of the British Embassy at the Court of St. Petersburg, &c. &c. &c. 8vo. pp. 363. London, Longman and Co. 1844.

"The Travelling Physician," now that he is established amongst us, seems resolved to write himself into public notice; and, were he to take a little more trouble in the arrangement and connection of his works, he would bid fair, we think, to attain considerable success in this way. He evidently possesses a good many of the qualities that are necessary for a popular medical writer. He has a lively and quick perception, a considerable fund of agreeable information, and an alert and active mind, that is ever on the search for whatever bears on his subject. With light and nimble wing, he skims the surface of a variety of topics; never rudely brushing his feathers against any impediment in his way, nor encountering, save in the most peaceful collision, another brother of the quill.

The great defect of the present work is the utter want of unity or method in its execution. The imp of confusion seems to have been perched on the pen of the author, whenever he took it into his hand; and we might even fancy that it had more than once maliciously displaced the pages of the manuscript, when the knight had left his *sanctum* to repose. At the end of a chapter, the reader will not unfrequently find himself in uncertainty whether he understands the drift or meaning of his author's reflections; nay, the thought may arise in his mind, has the author himself quite formed his opinion on the subject which he has undertaken to discuss?

There is moreover so much irrelevant matter introduced, and the various topics, that are brought under consideration, are held together by so flimsy a thread of connection, that we have often exclaimed in silence to ourselves, what has all this to do with an "Apology for the Nerves?" But, when we called to mind the motto of the work, *Without a nervous system there is no animal, there can be none; without a circulating one there are myriads*—a dogma, by the bye, the truth of which is merely asserted, and by no means proved—we perceived the train of our author's reasoning that whatever appertains, whether in health or in disease, to a living animal body, must have some relation or another with the *nerves*. Sir George shews his good judgment in never introducing the appellation of his work into the discussion of any of the topics which it treats of. The reader thus forgets the professed object of the volume; and, as he passes over its pages, may naturally suppose that he has got hold of some "Leaves out of a Physician's Scrap-book," or "Random Sketches in Medicine," dashed off with a free and easy pen to amuse a passing hour. That Sir George is capable of something of a higher cast than he has yet essayed, we have no doubt. He has been an observant man of the world; and there is no want of tact or cleverness about him, in distinguishing truth from error, and in applying his knowledge to practice. He is ever the courteous polite gentleman to all whom he may chance to mention, avoiding censure where he cannot praise, and always expressing his criticisms in the least presumptuous manner. We should suppose that he was much liked by his *confreres* at Petersburg, and we trust that he will experience from his brethren here the same good feeling that he ever seeks to shew to others.

After these remarks, we need scarcely say that, in reviewing such a work as the present, all that we can do is to select such passages as may either instruct by the novelty of their information, or interest by the manner in which this is conveyed: and first of the

*Effects of Extreme Cold in Russia.*

"I have witnessed the effects of cold too long endured upon the little postillions, who are barbarously exposed to it in the winter season at St. Petersburg. The lads bear it for a time, as they sit on their horses, clapping their hands, and singing to keep up their courage; but this fails them by degrees, and finally benumbed, they fall from their saddles in a state of torpor which nothing but rolling them in the snow will overcome. There is seldom a fête given at St. Petersburg, in the extreme cold weather, that occurrences of this sort are not recorded. In very cold nights the sentries are frequently frozen to death, if not relieved at short intervals.

"As long as nervous excitement can be kept up, the resistance of cold is very great. General Piroffsky informed me, that in the expedition to Khiva, notwithstanding the intenseness of the cold, the soldiers marched along singing, with the breasts of their coats open, but only as long as they were flushed with the hopes of success. Where there is nothing to excite, and where exposure to cold takes place under the common routine of parade, its depressing effects are lamentably felt by those long exposed to it. In the time of the Grand Duke Constantine, a regiment of horse was marched from Strelna to St. Petersburg, a distance of twelve miles and upwards. He marched at their head at a foot pace all the way. He had well wadded himself, and smeared his face over with oil. It was the gratification of a whim to expose the soldiers to a great degree of cold. They arrived at the square before the palace, and were dismissed to their barracks. The following day one-third of the regiment was in the hospital, attacked by nervous fever, of which many died. There was no stimulus of necessity in this case, but the moral feeling aggravated the physical suffering. The soldier is much better taken care of now-a-days in Russia. Cerebral affections are a consequence of reaction when the nervous system has been too much exhausted. I have mentioned elsewhere the case of the bishop of Nicolai, who died in a few hours of brain fever from exposing himself to severe cold during the performance of a religious rite." 21.

Passing over Chapters II. and III. on the Blood, Muscular Motion, Nutrition, and Secretion, we come to the fourth, which professes to treat of Sympathy, Phrenology, Mesmerism, Sleep and Dreams—a rather droll concatenation of subjects. The Chapter opens in a pleasant jocular mood, our author being no lover of the dry crumbs of philosophy. "When Falstaff said he knew the Prince by instinct, he solved a very difficult problem. We are often in the same predicament, without being able to extricate ourselves so satisfactorily as the worthy knight. Where ideas fail, we can sometimes, as Mephistopheles said to the student, substitute a sonorous word; at other times, ideas are more redundant than our means of expression.

"John Hunter has been criticised for his 'stimulus of necessity,' as an unmeaning term, yet there is something very instinctively intelligible in the idea it conveys.

"Some physiologists have denied sympathy, but have furnished us with no better word for a multitude of effects, which cannot be otherwise explained than by the use of some conventional term." 48.

The complicated phenomena of Sympathy, or, at least, the laws which regulate them, evidently constitute a subject that Sir George has no pleasure in grappling with. We are amused with the quiet way in which he disposes of the Excito-motory functions, and their clever expositor.

"Dr. Marshall Hall's view of the sympathies merits due attention, and must be read with great interest; but it is impossible to make works of this kind duly appreciated by a few extracts.

"The whole bears upon every part; but each single part conveys no accurate idea of the whole." 57.

The next topic discussed is—

*Phrenology.*

Sir George seems to be a hesitating believer in the doctrines not only of Gall and Spurzheim, but of Lavater also. He admits that there is still very great uncertainty as to the locality or material seat of certain faculties and passions, and takes the opportunity of quoting with approbation the shrewd remark of Mr. Travers, that "cerebral regions and cerebral agencies are as indispensable to the production of local physical sensations as to the operations of the mind. The phrenological system, I may here remark, owes its existence to the countenance which it derives from a twilight of truth, though only sufficient to serve as a beacon to the absurdities with which it is enveloped."—*Physiology of Inflammation.*

That the size of the Brain may become increased, even in an adult, in consequence of the unusual exercise of the mental powers, is a fact that has been asserted to have been observed in many instances. One of the most remarkable examples is that adduced by a writer in the *Dict. des Sciences Medicales.* "On peut en citer un exemple, le plus connu qu'on a observé, dans la personne de Napoleon, dont la tete, peu volumineuse dans sa jeunesse, avait acquis depuis quelques années un development presque enorme."

We have heard a good deal of late, in this metropolis, of the organ of Acquisitiveness in certain ladies gaining an undue ascendancy over that of their Conscientiousness and Love of Justice; but we were not aware, till we read the present volume, how endemic the malady has become amongst us. "There are few shop-keepers," says Sir George, "in London, who cannot point out persons of rank who will readily lay out £100.; yet, if they have an opportunity, will steal a yard of lace!" We hope that things are not quite so bad as this yet!

The following excellent remarks are appended, in the way of comment on a case of *cerebral excitement*, in which the opinion of Spurzheim had been followed with marked advantage.

"When in Edinburgh, I was clinical clerk to the late Dr. Rutherford, with whose memory are associated most grateful recollections, and from whom I

received most marked kindnesses. His practice was considered by the students, who know so much, or think they know so much, as puerile and inert, and his visits drew but few followers into the wards. Cases, which seemed to us to demand the most active treatment and a free use of the lancet, were treated by a saline diaphoretic mixture and a foot bath, and got well under such treatment. He once said to me, with a smile, my practice differs from that of my colleagues; but it is my object to let the students see how much Nature will do in many instances, and how patients recover under the most simple treatment, and by the removal only of all exciting causes. It is too common with you all to ascribe every thing to the medicines you administer. The very active treatment you employ relieves the symptoms, and you take great credit to yourselves for your decided practice; but you forget that long convalescences follow, and the constitution is shattered and impaired by such abstraction of its powers. You will find that patients who have been apparently cured by large bleedings which have conquered pain in the first instance, remain eventually longer in the wards than those who have not been so speedily relieved; moreover, you will find them return again, after their dismissal, with dropsy and chronic affections. As regarded bleeding, the Doctor never took away more than from eight to twelve ounces at a time; and now, after a period of twenty-five years, his views of things, and those of his colleague, Dr. Gregory, seem to have been correct; for, as the latter has beautifully expressed it, 'Nam ut sanguis semel missus nunquam in venas redit, sic neque vires cum illo amissæ in variis morbis unquam refici possunt.' " 67.

From Phrenology we pass on to—

*Mesmerism.*

Our author is too acute to believe in any of the mountebankeries —Clairvoyance for example—of this " forlorn thing," as Marshall Hall calls it. He mentions the case of a female impostor at St. Petersburg, who, among other feats, extracted living worms from the points of the fingers! As a matter of course, we have no intention of discussing the merits of the *science*, at present. Suffice it to say that the abstraction or concentration of the attention, on the part of the magnetised person, is sufficient to account for very many of the marvellous phenomena that have been so much talked of. Violent mental emotions will often render the body insensible to suffering. The most agonising tooth-ache generally ceases before the dentist has applied his instruments; a man with a wooden leg has been known to leap over a gate when a mad bull was coming down upon him; a paralytic patient has sprung out of bed on the alarm of his house being on fire; and many a soldier and sailor have looked, with incredulous astonishment, at the feats which they had accomplished on the preceding day, during the mad excitement of the battle. We adduce the following case—which came under our author's cognizance—as an instance not of a cure by Mesmerism, but of the ill effects of medical men (probably) doing too much in certain circumstances.

" A lady, the wife of a physician, met with the following accident:—Her foot having slipped in mounting the steps to her door, she fell down the area, and concussion of the spine was the consequence. She lost all power of motion in the lower extremities. Various means were resorted to: blisters, setons, frictions, with tartarized antimony, galvanism, and, finally, she suffered the excruciating tortures of seven moxas burnt upon the sacrum, at different periods,— all without effect. She remained a cripple, without any power of moving her limbs, and this for the space of twelve months. She was carried in a litter to the steam-boat, which took her to Paris by Havre. Upon here arrival there she was treated by Recamier. He introduced a series of setons from the nape of the neck to the sacrum, but with no better effect. She was a woman of great moral courage. *Nil desperandum.* She resolved on trying magnetism. A female treated her, and in six weeks she was going the rounds of a gay Parisian life, with her limbs perfectly restored." 73.

Such instances ought to suggest a useful admonition to medical men against the over-officious employment of unpleasant and painful remedies in cases of chronic functional disturbance. In such a case as the preceding, electro-magnetism has been used of late years with very marked benefit.

Passing over our author's remarks on Sleep, Dreams, and the senses of Vision, Hearing, Taste, and Smell, we come to the following remarks, under the head of the sense of Touch, on *the good effects of rubbing the skin!*

"There is, I believe, a great deal to be done, in a medical sense, by mere rubbing, if well performed. In many instances, it is the best means of soothing, and induces sleep as sure as an opiate. In many female obstructions, constant rubbing with the flesh-brush will do more than steel and myrrh pills; but this must not be trusted to the patient, for then it is never done effectually, either from forgetfulness, fatigue, or the intervention of a hundred other causes. A steady old nurse should perform this operation. In the mesenteric affections of children, the same system is most efficacious. In chronic diarrhœa I have found it most beneficial; and in that half-and-half kind of gout which sometimes makes its appearance, and threatens worse attacks for the future, this will often effect wonders."

"A Gentleman complained to me, that he had had an attack of the common cholera some months previously, and that his bowels had since been in so disordered a state as to interfere with his pursuits, thus being a source of great annoyance to him, and rendering him irritable. I told him to buy a flesh brush, or a pair of hair gloves, and rub himself night and morning. He told me, some weeks afterwards, that he had found the greatest benefit from the rubbing system.

"It is questionable if the hair shirt of catholic penance might not be useful in some of these chronic cases of diarrhœa." 107.

There is a long and rambling Chapter on *Headaches.* It contains, however, some very shrewd remarks, which our readers may thank us for picking out for their use. In those *nervous* headaches, which are accompanied with excessive irritability of mind, and waywardness of temper—perhaps even with threatenings of mental aberration—Sir George very judiciously recommends the steady use of the shower-bath as one of the best of remedies.

If the attacks of pain be periodic, Arsenic is generally useful. When Quinine is administered, it should be combined with an aperient; "for an open state of the bowels is always a source of relief, although purgatives do but little good, if given alone." The well-known Griffith's mixture, with a blue pill once or twice a week, produces, in many cases, excellent effects in weak and debilitated habits. Valerian and Hoffman's drops will often give temporary relief.

"In the rheumatic headache we have something more tangible. If we do not succeed in relieving it, we seem to set about the task with more confidence. It is the most painful perhaps of all the kinds, but it is sometimes remediable. It often begins in a point over the eye, or in the temple, and then gains the whole head. A full dose of colchicum, at the commencement, will sometimes cut it off, and with this a dose of calomel and opium at night. A lady whom I treated for this affection, was relieved by a mixture of decoction of bark and guaiacum, in equal parts, of which she took a dose three times a day. With her it proved a specific, and she wrote to me for the prescription sometime afterwards from Italy, as she had found nothing else relieve her." 142.

In the Hæmorrhoidal form of the complaint, leeches to the anus are, says our author, a specific. *La tete est degagée* is the expression of all who are thus treated. In the headaches of children, in whom there is any predisposition to Hydrocephalus, the application of cold to the head, frequent blistering behind the ears, or the insertion of a seton in one of the arms, are the most potent of all remedies.

The seventh Chapter is occupied with cursory remarks on many nervous diseases—*Epilepsy, Hysteria, Palsy, Catalepsy, Hydrophobia, Trismus, Delirium tremens, Pertussis,* and *Chorea*. We select the following, as favourable specimens of the general manner in which these subjects are treated.

*Hooping Cough.*

"I may here mention the treatment which we find most useful in northern climates. We have not the advantage of changing the air for a long period, so that the complaint is often of formidable duration; but as soon as the febrile stage has subsided, which generally takes place towards the end of the third week, doses of musk seem to have a specific influence. I have seen the most happy effects from its use, both in the general practice of my colleagues, and my own, in St. Petersburg. A grain of musk three or four times a-day, will, in general, arrest the most convulsive species of coughing in a few days.

"I have generally, in the early stages, experienced great advantage from the application of leeches to the temples; for I believe that there is very often congestion in the brain, and that the latter, if not previously, is often secondarily, affected." 178.

*Chorea.*

"Dr. Depp, chief physician to the Foundling Hospital in St. Petersburg, acquired a great and deserved reputation for his success in the treatment of this disease.

"His plan was almost wholly confined to the use of the shower-bath at all seasons of the year; and as soon as the weather permitted, he removed his patients into the country, selecting as elevated a position as possible, that they might enjoy the advantages of a bracing atmosphere. He insisted also upon the hair being cut close, and the head spunged with cold vinegar and water at several periods during the day; the shower bath to be employed as soon as the patient left the bed, or rather hard mattress, which he also insisted upon. He prescribed few medicines, and nothing usurping the name of a specific. The bowels were to be regulated by the mildest purgatives, but the shower-bath seemed to supersede the necessity of these.

"I saw but few cases of this singular affection, but a very aggravated one was most materially relieved by this treatment." 180.

We cannot answer for the results of medical practice in Russia; but of this we feel assured, that no judicious medical man in this country will trust to the use of cold bathing for the cure of Chorea. The internal exhibition of steel—and in some cases of Arsenic—is with us very generally necessary.

Chapter VIII. touches lightly upon *Cholera—Scorbutus—Scurvy* (how comes this to be treated apart from its Latin synonym ?), and *Diabetes*—a somewhat incongruous conjunction, and this too in a work that professes to treat of the nerves!

Chapter IX. is devoted to the consideration of *Fevers*, or rather of the ætiological question, Is the primary morbid impression on the blood, or on the nerves, in febrile diseases ? Sir George, as we might anticipate, leans to the latter opinion, and quotes a variety of passages from the writings of Billing, Copland, Sir Alex. Crichton, and others in favour of his own views. The following sensible observations, on the predisposing influence of the depressing emotions, may be aptly quoted.

" The dread of evil is sometimes greater than the evil itself. Of this the cholera afforded us a good illustration. When it was raging at Moscow, we of the faculty greeted each other in the streets of St. Petersburg with very lengthened faces; but as soon as it came among us, and we plunged in *medias res*, there was no fear apparent. The stimulus of exertion to vie with each other in finding some remedial means, became a buckler to ourselves.

" The fever in Edinburgh in 1818, to which I have alluded, afforded a very striking evidence of the effects of moral depression upon one individual, who was superintendent of the Queensberry House Fever Hospital. He was the most active man in the institution, and escaped the fever, when all the medical inmates, myself among others, had passed through the ordeal. He seemed proof against its influence. But he wrote a book upon the subject,—a large volume, and embarked all his little means in the speculation. It turned out unfortunately for him, and preyed upon his mind. He took the fever when the wards were almost cleared of it, and, though he struggled through it, he died of its consequences.

" I think that such information as the following has saddened the heart of many, upon inquiry after old acquaintances :—' He got wrong in his affairs, was harassed by domestic misfortunes, got into a low nervous state, caught cold, which terminated in fever, of which he died.' " 219.

Our author, following in the footsteps of Dr. Macculloch, attributes a great variety of anomalous and indistinctly-marked maladies to the influence of Malaria. What is commonly called *ill-health*—without the specific symptoms of any one disease—may very often, he thinks, be traced to the operation of this cause. This remark is especially applicable to medical practice in the Russian metropolis.

" Situated in a bog, surrounded by marsh and peaty formations on every side, the city of St. Petersburg rises a monument of the triumph of art over nature. It was as great a feat for Peter the Great to erect his capital in such a situation, as for the Roman emperor to conquer the sea by his bridges. It was determined by the savants in Paris, when they discussed the causes of the *black death*, that if the disease had occurred in Sardinia, not a soul would have been left alive, so much did they attribute to local influence in the creation of that epidemic; and were it not for the hard frosts in winter, malaria would probably destroy the population of St. Petersburg."

" There are few females in St. Petersburg who are not subject to nervous headaches. These are, I think, attributable in a great measure to the heat of the rooms and the close air of the apartments, which are useful only as preventatives of phthisis, but are far from conducive to strong health; these affections of the head are accompanied by varieties of uneasy feelings, loss of appetite, sleeplessness, vertigo, and more or less of fever. They are not relieved by country air nor the admission of free air into the houses, nor by the removal of the double windows at this season, because with the circumstances that permit of these operations, others arise. The emanations from the decayed vegetable matter which were kept under by a coat of snow and hard frost are now let loose, and it is in the spring season that all these affections are most aggravated. This is the season of the greatest mortality; and the breaking up of the ice and its departure from the Neva, are most dreaded by those who have long been ill. Il s'en ira avec le debacle, is a phrase in the mouths of all the Mrs. Quicklys in St. Petersburg. It is the turning of the tide with them. To those, however, who are able to get away, it is the speedy return to pristine health. Those who are robust by nature brave this climate with impunity; those whose lives are active, and employments sufficient to occupy their time, enjoy the best of health; but, to the ailing and nervous, it is a species of martyrdom. Hypochondriacs abound, and I have known such quit the country for fear of worse consequences in a mental sense, and return again in a few months to laugh at their own folly." 234.

It would seem, from our author's experience, that the diseases of St. Petersburg—even those of a decidedly inflammatory character—do not bear well very copious, or frequently-repeated, depletions of blood. Under the most favourable circumstances, the convalescence from any serious illness is apt to be exceedingly tedious *there*, more especially during the winter months. To re-establish the health, it is very generally necessary to leave the banks of the Neva for some more healthy locality, on the breaking up of the ice in the Gulf of Finland.

In the treatment of *Agues*, Sir George lays much stress on the importance of administering one or two grains of calomel, for two or three nights successively before, and also during, the use of the bark or quinine. The mercurial seems decidedly to promote the anti-periodic virtues of the latter. The Muriate of Ammonia also has been found by our author a very useful adjuvant in many cases. His formula is this:

℞ Ammon. muriatis ʒj.

Extract. Taraxaci ℥ss.

Aquæ petroselin. ℥vij. M.

Cujus sumat æger coch. duo ampla ter die.

We need scarcely say, that *Rheumatic* and *Neuralgic* complaints are exceedingly common in St. Petersburg. "Under all circumstances, they are very tedious, and, in general, perfect recovery is not effected till the season of migration arrives."

Chapter XII. professes to give us an account of *Some Peculiarities of German Practice*—"which," we are informed, "holds a middle rank of action between the French and the English, being more energetic than the former and less so than the latter." We have been much disappointed with this Chapter, as we had quite expected from its title—reasonably too, it will be admitted—to have found on perusal a good deal of interesting and even instructive matter. We select one or two passages that are most worthy of notice.

*Grape Cure.*

"Those who have practised long in Russia will have been made conversant with the *cure du raisin.* I had an opportunity of becoming so when in the south of the empire, and in a grape country. It is necessary to state in what this cure consists, and for what class of diseases it is recommended. The latter may be dismissed at once, by stating that all those functional nervous affections, which resist the routine of treatment generally employed, are the cases which may be so benefited, seeing that the discipline is more intolerable than the disorders for which it is instituted. A lady of rank leaves her bed of down and cushioned canopy, and migrates into the country,—turns a poor family out of their habitation (not without making them an ample recompense) and becomes the tenant of a filthy hut. This is part of the cure, viz. to forego all luxury, to sleep in the peasant's crib, to sit upon his bench, and to avoid anything in the shape of comfort. The grape alone for meat—the grape for drink; a small quantity of dry bread is perhaps allowed. This is continued for the space of three weeks, and it is no wonder, if all circumstances are taken into consideration, that a cure is effected. I have known people of the highest rank subject themselves to such discipline, and have full faith in its results. It is homœopathy and hydropathy in another shape, and as the Italians say of all the varieties of form in which they make their pastes, c'est *toujours du macaroni.*" 282.

*A New Vesicatory.*

"There is an Epispastic much used abroad, which I think would be advantageously introduced into British practice. It is the 'Pommade vegetale de Swisse,' a preparation of the bark of the mezereon, which, when rubbed on the skin, produces immediate vesication, without causing the same irritation, and never accompanied by any of the unpleasant symptoms which are sometimes produced by the blistering fly. It is employed generally to produce vesication behind the ears, and particularly in the affection we are speaking of. A small portion, rubbed strongly in, raises a blister in a few hours, and the surface may be made to discharge for any length of time, by smearing it over with the same preparation. Where a large blister of the lytta has been first applied, this preparation answers much better than the savine ointment in keeping it open. I have not been able to procure it in any of the druggist or patent medicine shops in this metropolis. In cases threatening hydrocephalus, the Germans apply it behind the ears, and keep one side in a state of exudation for years consecutively, where they do not insert an issue." 143.

On more than one occasion, Sir George states that, in his Russian practice, the ordinary *blue pill* did not seem to exercise its usual effects on the system, either in visceral affections or in the venereal disease. In the treatment of the former, calomel, and in that of the latter, the bichloride was greatly preferable. It is to be remarked that the *blue pill,* which was used by him, was imported direct from London.

We must here remind Sir George, that an author is not entitled to copy, even from his own writings, without giving some intimation to his readers. Not a few passages in this Chapter are merely reprints of what has already been made known in his "Essay on Thermal Comfort"—published, too, within the last twelve months! But we suppose that we must not press hard upon any one, who gets up new books so quickly as the "Apology" has obviously been.

It only remains to notice the Appendix; and, in doing this, we must confess that we have rarely met with such a *melée* of discordant articles jumbled together in the most admired confusion. First of all comes one on the Venereal Disease; and this is followed by others on Cancer, the Cæsarian Operation, Nerven-Schlag, Disease of the Kidney, Poisons, Rupture of the Gall-bladder, Puncture of the Intestines, Mesmerism (a second time), Experiments on Animals, Sound, and the *Plica Polonica.* On this last-named subject, we had certainly anticipated to have found something novel and instructive; but the truth is, that Sir George seems to be quite as much puzzled to know what to say about it, as any one who has never crossed the Channel. Hear his droll confession: "To unfold a plain unvarnished tale, must be left, I believe, to the Ghost of Hamlet; for no one living seems disposed to do it. We find that not only the real existence of the disease is a matter of contention, but its origin and progress are equally twisted, like itself, from the path of truth by those who believe in its existence." All that can be learned from our author is that, in his opinion, it is a critical excretion from the scalp and roots of the hair, and is the natural curative process of a variety of complaints. The lower orders of the Polish population often encourage its formation and continuance,—by keeping the head hot and nasty—in obstinate cases of Ophthalmia, Rheumatic Headache and so forth. But it is unnecessary to extend our remarks; "as," to use Sir George's own words, "few describe what they themselves have seen, or the results of their own study and observation."

